# Biomass Increases Go under Cover: Woody Vegetation Dynamics in South African Rangelands

**DOI:** 10.1371/journal.pone.0127093

**Published:** 2015-05-13

**Authors:** Penelope J. Mograbi, Barend F. N. Erasmus, E. T. F. Witkowski, Gregory P. Asner, Konrad J. Wessels, Renaud Mathieu, David E. Knapp, Roberta E. Martin, Russell Main

**Affiliations:** 1 Restoration and Conservation Biology Research Group, School of Animal, Plant & Environmental Sciences, University of the Witwatersrand, Johannesburg, South Africa; 2 Centre for African Ecology, School of Animal, Plant & Environmental Sciences, University of the Witwatersrand, Johannesburg, South Africa; 3 Global Change and Sustainability Research Institute, University of the Witwatersrand, Johannesburg, South Africa; 4 Department of Global Ecology, Carnegie Institution for Science, Stanford, CA, United States of America; 5 Remote Sensing Research Unit, Council for Scientific and Industrial Research (CSIR)-Meraka Institute, Pretoria, South Africa; 6 Ecosystems Earth Observations, Natural Resources & Environment, Council for Scientific and Industrial Research (CSIR), Pretoria, South Africa; 7 University of Pretoria, Department of Geography, Geomatics, and Meteorology, Pretoria, South Africa; National University of Mongolia, MONGOLIA

## Abstract

Woody biomass dynamics are an expression of ecosystem function, yet biomass estimates do not provide information on the spatial distribution of woody vegetation within the vertical vegetation subcanopy. We demonstrate the ability of airborne light detection and ranging (LiDAR) to measure aboveground biomass and subcanopy structure, as an explanatory tool to unravel vegetation dynamics in structurally heterogeneous landscapes. We sampled three communal rangelands in Bushbuckridge, South Africa, utilised by rural communities for fuelwood harvesting. Woody biomass estimates ranged between 9 Mg ha^-1^ on gabbro geology sites to 27 Mg ha^-1^ on granitic geology sites. Despite predictions of woodland depletion due to unsustainable fuelwood extraction in previous studies, biomass in all the communal rangelands increased between 2008 and 2012. Annual biomass productivity estimates (10–14% p.a.) were higher than previous estimates of 4% and likely a significant contributor to the previous underestimations of modelled biomass supply. We show that biomass increases are attributable to growth of vegetation <5 m in height, and that, in the high wood extraction rangeland, 79% of the changes in the vertical vegetation subcanopy are gains in the 1-3m height class. The higher the wood extraction pressure on the rangelands, the greater the biomass increases in the low height classes within the subcanopy, likely a strong resprouting response to intensive harvesting. Yet, fuelwood shortages are still occurring, as evidenced by the losses in the tall tree height class in the high extraction rangeland. Loss of large trees and gain in subcanopy shrubs could result in a structurally simple landscape with reduced functional capacity. This research demonstrates that intensive harvesting can, paradoxically, increase biomass and this has implications for the sustainability of ecosystem service provision. The structural implications of biomass increases in communal rangelands could be misinterpreted as woodland recovery in the absence of three-dimensional, subcanopy information.

## Introduction

Woody biomass is a fundamental expression of terrestrial ecosystem functioning, (e.g. primary productivity, land-atmosphere gas exchange and nutrient regulation), and can be used for the quantification of ecosystem services, such as fuelwood and carbon sequestration. Biomass distribution reflects the spatial pattern of topo-edaphic and climatic gradients [1–3] and responses to disturbance [4–7]. However, biomass estimation remains challenging, particularly in environments with highly variable species composition and structural complexity [8–10].

Savannas, as complex tree-grass ecosystems, are structurally heterogeneous and are best described by three-dimensional metrics [11]. As such, savannas are ideal for examining the biomass dynamics in structurally complex vegetation. While total precipitation sets the upper boundaries on woody cover in savannas [12], their ‘woody cover potential’ is often unrealised [13–14] as a result of disturbances, such as fire [15–19] and herbivory [20–22]. A major driver in savanna ecosystem structure and function is the influence of people on the landscape [15,23], particularly through natural resource use, such as fuelwood harvesting [24]. Yet, the contributions of anthropogenic changes to savanna biomass dynamics are poorly understood.

Millions of people in Africa rely on woody vegetation for energy, extracted from both communal [25–27] and protected areas [28–29]. Within southern Africa, South Africa has a high per-capita use of fuelwood as a primary energy supply; despite having substantial access to electricity (66% of national population) [30]. Within this context, 93% of current fuelwood demands are no longer met by collection of dead wood [31]. Thus, live wood harvesting occurs around settlements and is a major driving force in woodland degradation in semi-arid ecosystems in southern Africa, particularly in the South African Lowveld (low altitude) savannas [7,24,32]. This is concerning because localised fuelwood scarcity is already being experienced, and the situation is unlikely to improve in the future [33]. Indeed, localised fuelwood shortages have facilitated the development of fuelwood markets [34–35], effectively increasing the harvestable area and thus the impacts of fuelwood extraction may become less of a localised phenomenon. Despite fuelwood markets contributing to rural livelihoods [34–35], they have the unfortunate knock-on effect of artificially maintaining perceptions of fuelwood abundance [36]. Although a depletion of woodland biomass was predicted to occur in Bushbuckridge, South Africa, by 2011 [24] and more recently, by 2024, at current extraction rates [32], the interactions between socioeconomic and environmental factors driving natural resource use are complex, non-linear systems that are difficult to quantify [37]. However, the above predictions do raise the concern that woody vegetation harvesting, driven by increased demand and greater extraction amounts is unsustainable [38] and reduces the ability of ecosystems to provide ecosystem goods and services, fuelling the link between rural poverty and environmental impoverishment [39].

Wood harvesting changes not only biomass, but also vertical stratification of vegetation. Vertical vegetation complexity has relevance to ecosystem function as canopy height is related to biomass and productivity [40], biodiversity [41–43] and contributes to structural heterogeneity [44]. We submit that a method of understanding and, potentially, improving biomass change estimations, is to examine the vertical vegetation structure. We believe that by observing the interplay between woody biomass change and subcanopy structural change, drivers of biomass dynamics may be revealed.

Vertical subcanopy structure of vegetation canopies, however, cannot be derived from traditional two-dimensional remote sensing methods and top of canopy cover is a poor predictor of subcanopy cover [45]; three-dimensional (3-D) field-based efforts are impractical at landscape scales. Light detection and ranging (LiDAR) is a valuable tool for repeat estimation and monitoring of biomass, whilst providing subcanopy information, over large geographic areas and with fine-scale detail [46]. Repeat LiDAR campaigns have enabled tracking of woody biomass change as well as variation in the 3-D structure of the vegetation, providing the means to test previous fuelwood supply-demand model predictions [24,32], and to make inferences about the sustainability of wood provision under continued wood extraction pressure. The aim of this research is to utilize the power of airborne LiDAR to assess changes in aboveground biomass and subcanopy structure, as a unique window into unravelling vegetation dynamics in structurally heterogeneous landscapes.

## Methods

### Study Site

Permission to conduct fieldwork in the Bushbuckridge communal rangelands was granted by the local headmen. This study is part of a broad, long-standing relationship with the local community and the University of the Witwatersrand to conduct ecological research in their communal land. The field studies did not involve endangered or protected species. The study sites were located within the Bushbuckridge Municipality in the Lowveld region, a semi-arid savanna in South Africa. Summer rainfall (October to May) usually falls in convective thunderstorms and ranges between >900 mm per annum in the west and 500 mm per annum in the east with an mean annual precipitation (MAP) coefficient of variation of 25%. Summers are hot and humid with mean daily maxima of 30°C and winters are mild and dry with mean daily maxima of 23°C. Droughts can be prolonged and may be experienced every ten years. Within the timeframe of this study (2008–2012), the 2006–2007 and 2007–2008 summer rainfall was below average and the 2011–2012 was a particularly wet summer. Within seasons, notable rainfall peaks occurred in April 2010 (4.1-fold more rain than the monthly 8-year average) and January 2012 (2.4-fold higher than the monthly 8-year average).

The terrain is shallowly undulating and the geology is dominated by granite with local Timbavati gabbro intrusions. Classic catenal sequences are common in areas with shallow, sandy, dystrophic soils on the uplands and deeper, clayey, eutrophic soils on the bottom slopes [7]. The predominant vegetation type is granite lowveld, but the region also contains gabbro grassy bushveld and legogote sour bushveld [47]. Common plant species on the granite Lowveld uplands include: *Terminalia sericea*, *Combretum zeyheri* and *C*. *apiculatum*; the bottom slopes are characterised by *Acacia nigrescens*, *Dichrostachys cinerea* and *Grewia bicolor* [47]. Other frequently occurring species are *Sclerocarya birrea*, *Lannea schweinfurthii*, *Ziziphus mucronata*, *Dalbergia melanoxylon*, *Peltophorum africanum and Pterocarpus rotundifolius*. The majority of the woody biomass in the region is formed from *S*. *birrea*, *Pterocarpus angolensis* and *A*. *nigrescens* [7].

Bushbuckridge is surrounded by conservation land (both state-owned and private) [48] which increases the pressure for grazing and harvesting outside of protected areas. An overgrazing land-use legacy exists from intensively stocked, white-owned cattle farms from 1913 onwards [49]. Apartheid followed in 1948, with the Promotion of Bantu Self-Government Act of 1959, which forced black South Africans to live in ‘homelands’ [49]—centralised settlements on farms of 1000–2000 ha. Bushbuckridge Municipality was formed from the joining of Mhala in Gazankulu and Mpulaneng in Lebowa [2], with settlement boundaries defined by the old cadastral borders of the historical cattle ranches [50]. Although Bushbuckridge falls under state control, there is customary communal land tenure controlled by headmen who zone the land into residential, arable and communal areas for grazing of livestock and collection of timber and non-timber products (e.g. thatch, fruit, medicine) [51]. The settlements range from small, isolated villages to larger, dense settlements along major roads [33]. Human population density sharply increased between 1972 and 1994 to approximately 300 people/km^2^ [49] but these growth rates have declined over the past ten years [35]. Commensurate with human population growth in the area, the spatial footprint of the residential regions has expanded [37,52]. A foreboding of this decline was an observed reduction in the size-class distribution of the woodland vegetation with increasing distance from certain settlements [53].

**Fig 1:**
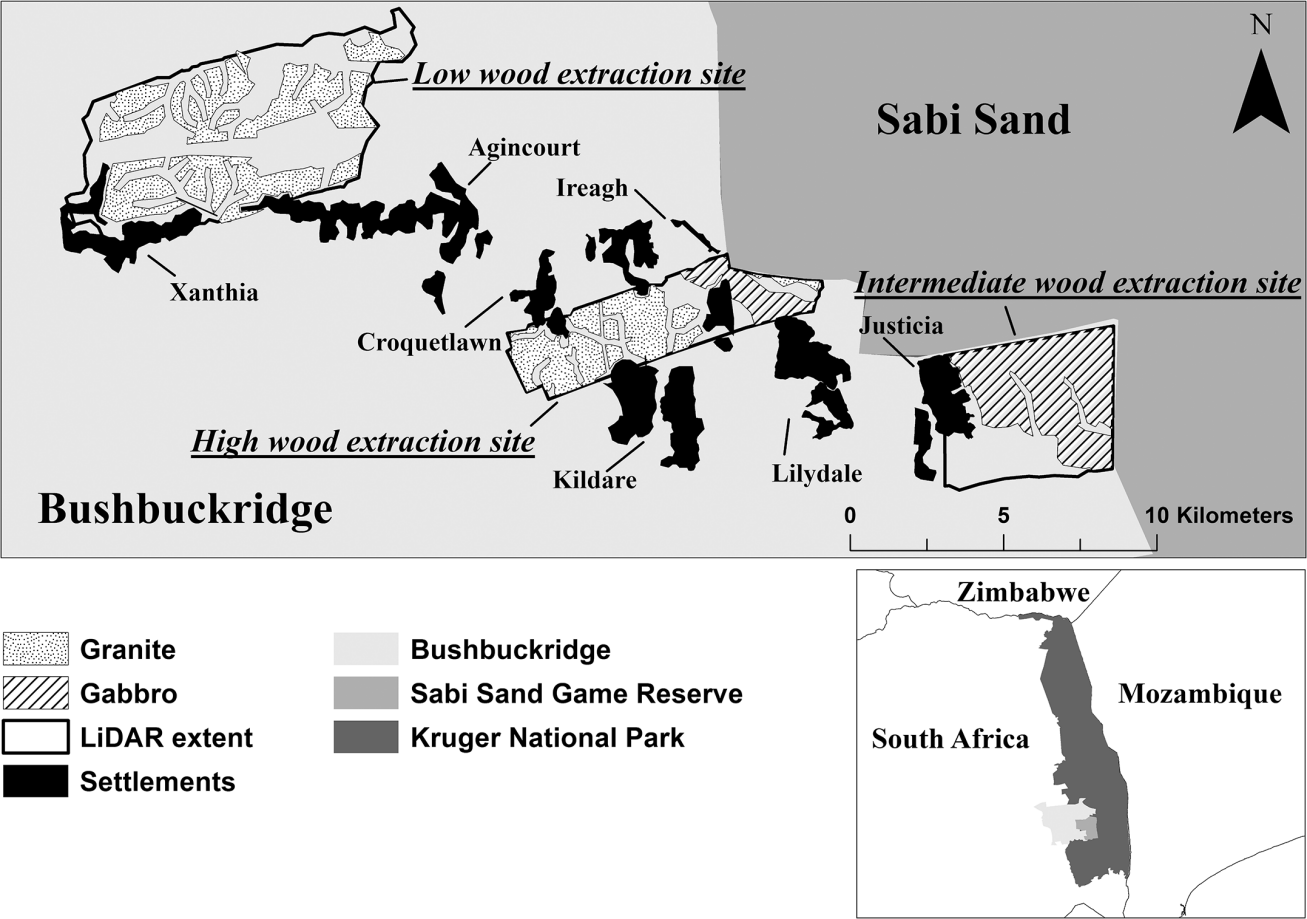
Study sites in Bushbuckridge municipality, located in the South African Lowveld. Sites are classified (from west to east) as low, high and intermediate wood extraction pressure based on the number of households and people utilising each rangeland. Settlements that utilise each rangeland are shown, including the names of the major settlements, as well as the location of the gabbro intrusions in the predominantly granitic landscape.

Within Bushbuckridge, three communal rangelands were chosen to represent different levels of natural resource utilisation. These rangelands are zoned for use by the following villages: Justicia; Croquetlawn, Ireagh and Kildare; Xanthia and Agincourt (Fig 1). The rangelands were classified according to the relative wood extraction pressure assessed using 2008 data on the number of people and households accessing a given rangeland and relative to this corresponding rangeland area: high (9.2 people ha^-1^, 1.56 households ha^-1^; using 2155 ha of rangeland); intermediate (1.8 people ha^-1^, 0.35 households ha^-1^; using 1815 ha of rangeland); and low (0.21 people ha^-1^, 0.04 households ha^-1^; using 4425 ha of rangeland) (see [53] for detailed demographic data). Although each rangeland is used by its corresponding settlements, use is not exclusive to these villages and foreigners (both local and cross-border immigrants) are known to harvest from these areas [38]. The intermediate-use intensity rangeland (Justicia) is the only example of exclusive access, as it is fenced on two sides by private conservation land and its location makes it more difficult to access from other villages [32].

### Field-derived biomass estimates

All field data were collected concurrently with the airborne LiDAR campaigns in April 2012. Field-plots (total n = 56; high extraction site n = 16; intermediate extraction site n = 20; low extraction site n = 20) of 25 m x 25 m were established within the extent of the communal rangelands LiDAR coverage, and their locations recorded with a differential Global Positioning System (Trimble GeoXH Handheld GPS). All heights and basal stem diameters on stems thicker than 5 cm on trees taller than 1.5 m in height were recorded. A ‘tree’ may refer to a single-stemmed or multi-stemmed individual derived from the same rootstock, whilst ‘stem’ refers to the all branches derived from a single point on the ground. These height and basal stem diameter field data were used to estimate field biomass using allometric relationships from Colgan et al. [9], an extensive harvesting study with the same woody species composition as Bushbuckridge, in the form:

$$m=0.109D^{\left(1.39+0.14\;\text{ln}(D)\right)}H^{0.73}\rho^{0.80}$$

where *m* is dry aboveground stem mass (kg), *D* is stem diameter (cm), *H* is height (m) and *ρ* is a unitless wood-specific gravity constant. The individual stem masses where then summed within each 25 m x 25 m plot to obtain plot-level field biomass, reported in Mg ha^-1^.

### Light detection and ranging (LiDAR) data

The communal rangelands were surveyed with airborne laser mapping as part of a Carnegie Airborne Observatory (http://cao.ciw.edu/) campaign in April 2008 and April 2012, concurrently with the collected fieldwork data in 2012. Small footprint, discrete-return LiDAR is a remote sensing method which estimates 3-D vegetation structure over large areas. The 2008 LiDAR data were collected from 2 000 m a.s.l. with the CAO-Alpha system with a laser pulse repetition frequency of 50 kHz and laser spot spacing of 1.1 m (see [54]); the 2012 data were collected with CAO-2 AToMS with a laser pulse repetition of 100 kHz and laser spot spacing of 1m (see [55]). The LiDAR system also provides accurate geo-locational information generated by a high performance inertial management unit (IMU) and global positioning system (GPS) [54]. The LiDAR product is a 3-D point cloud from which a canopy height model (CHM) was constructed from the difference between the digital terrain model (DTM, interpolated from the last LiDAR returns) and the digital surface model (DSM, interpolated from the first LiDAR returns). Spatial errors on the more coarse of the two products (2008 data) were <0.20 m vertically and <0.36m horizontally [54]. Although different sensors and processing methods were used for the 2008 and 2012 data, errors between corresponding DTM’S were <15cm.

Volumetric pixels (voxels) are formed by aggregating LiDAR laser returns into 1 m height classes [56]. The position of each voxel is taken from the voxel centroid relative to the ground. LiDAR return frequency, within each voxel, are reported as a percentage relative to the total number of LiDAR points in the complete vertical column, including the ground returns. These data are used to quantify subcanopy (i.e. vegetation beneath the canopy cover) structure.

### LiDAR-derived biomass estimates

LiDAR-derived metrics of woody vegetation can be used to estimate allometric relationships and infer biomass [2,8,9,32,57–58]. We derived a biomass regression model according to previously established methods by correlating the plot-level field-allometry and a corresponding LiDAR-derived H x CC (height x canopy cover) predictor metric calculated for each 25 m x 25 m grid cell created to correspond to the 25 m x 25 m field plots; H is plot-averaged (mean pixel height values >1.5 m) and CC is the proportion of canopy cover per plot (proportion of pixels >1.5 m in height). Both values were extracted from the CHM (see [9] for details). The H x CC metric is not only ecologically meaningful as it is an approximation of wood volume, but it also gives the best results over more complex metrics [2]. The height mask (>1.5 m) was used to account for the possibility of ground and tall grass being misclassified as vegetation. The LiDAR-derived predictor metrics were trained against field-derived biomass for each rangeland as they all exhibit different vegetation structural patterns, resulting from variable rainfall, different geologies and wood extraction pressures. Not only were these site-specific models able to explain more variation than one general equation; they were also deemed more ecologically valid. Biomass maps were then created by applying the site-specific biomass models to the LiDAR CHM extent (masked at heights >1.5 m) for each rangeland for both 2008 and 2012. Only grid cells that fit the criteria of an average height of >1.5 m (once pixels of <1.5 m were excluded) were used to estimate biomass as this is the vegetation that the fieldwork included. However, the cells that matched these criteria varied in both number and spatial location between 2008 and 2012. For the purposes of biomass change detection, only those cells that met the average height criteria for both years in the same location were considered. Riparian areas adjacent to streams in the rangelands were excluded from the biomass maps as they require separate calibration [2]. Similarly, cultivated fields and built-up areas were excluded.

### LiDAR-derived subcanopy analysis

The voxel data (5 m x 5 m x 1 m) were resampled to 25 m x 25 m x 1 m, making the data comparable to the biomass grid cell sizes, and stacked into the following ecologically relevant, vertical height classes: 1–3 m (shrubs and small trees in the ‘fire trap’ [16]); 3–5 m (trees in the ‘elephant trap’ [22]); 5–10 m (tall trees contribute to structural diversity and thus to ecosystem function [59]); >10 m (very tall trees, ‘keystone structures’ [60], are often culturally important trees conserved in the rangelands [61]). These data were used to detect changes in the distribution of the vegetation size classes within the vertical vegetation column. “LiDAR returns” refers the percentage of laser pulses that were emitted from the sensor, hit an object and returned to the sensor. In the results, “Total % LiDAR returns” refers to the returns for the full vegetation column—excluding the ground returns. “% Subcanopy returns” refers to the LiDAR hits within a particular height category. Higher subcanopy returns implies greater density of vegetation in that height class.

### Data extraction and analysis

Features of the settlements (e.g. roads, villages, crop fields) and rivers were manually digitised using a combination of SPOT 5 imagery (panchromatic-multispectral merge (480–890 nm), 2.5 m spatial resolution, www.spotimage.com) and aerial photographs (50 cm resolution, www.ngi.gov.za). Biomass estimates were extracted from the maximum number of randomly distributed points with a minimum enforced distance of 50 m to avoid spatial autocorrelation, based on the results of semivariograms (calculated in ENVI v4.7). All data were analysed in R v3.0 (R Core Team), including descriptive statistics, linear regression models and correlations. Biomass estimates were tested with Shapiro-Wilk Normality tests from the “fBasics” package and all sites in both 2008 and 2012 were found to be non-normally distributed (p < 0.001). Thus, a non-parametric Wilcoxon rank sum test was used to analyse differences between means over time within sites.

## Results

### Biomass models

A strong relationship existed between the field allometry and LiDAR metrics, although the highly heterogeneous rangeland resulted in high root mean square error (RMSE) values in both high and low use sites on granitic substrates (18.6 and 19.1 Mg ha^-1^, respectively) (Table 1). The increase in variability with increase in biomass indicated (S1 Fig) less agreement between the field allometry and LiDAR metrics at higher biomass values. This is a common phenomenon, termed ‘heteroskedasticity’, of model performance at higher biomass levels where the error variance is not consistent over all the observations [62]. Most typically, modelling the error structure shows a fanning pattern of increasing variance with increasing biomass [62], and this is true of the residual structure for both the high and low wood extraction sites (S1 Fig).

**Table 1:** Site-specific biomass models derived from field allometry and LiDAR metric linear regression.

Extraction pressure	Model	R^2^	n	RMSE (Mg ha^-1^)
**high**	y = 2312.3x - 157.14	0.78	16	18.6
**intermediate**	y = 409.57x + 252.74	0.60	20	4.8
**low**	y = 913.9x + 127.86	0.68	20	19.1

In the model equations, y refers to the plot-level (25 m x 25 m) biomass estimate (kg/625 m^2^) and x to the LiDAR-derived H x CC predictor metrics, where H is plot-averaged height (> 1.5 m) and CC is the proportion of canopy cover (> 1.5 m in height) per plot. Root mean square error (RMSE) was reported in Mg ha^-1^ for ease of interpretation and n is number of 25 m x 25 m plots.

### Biomass dynamics

Mean biomass (± SD) in 2008 at the high, intermediate and low extraction sites was: 26.99 ± 16.43 Mg ha^-1^ (n = 102 cells), 9.42 ± 4.13 Mg ha^-1^ (n = 291 cells), and 21.18 ± 12.04 Mg ha^-1^ (n = 1654 cells), respectively. Biomass increased significantly at all sites between 2008 and 2012 by an average 18.38 Mg ha^-1^ (highest use site: W = 3036, p <0.001), 5.45 Mg ha^-1^ (intermediate use site: W = 16780, p <0.001), and 11.34 Mg ha^-1^ (low use site: W = 771641, p <0.001) (Table 2).

**Table 2:** Mean biomass increase (Mg ha^-1^) at sites under varying wood extraction pressures.

	Extraction pressure		
	High (n = 102)	Intermediate (n = 291)	Low (n = 1654)
**2008 (mean ± S.D.)**	26.99 ± 16.43	9.42 ± 4.13	21.18 ± 12.04
**2012 (mean ± S.D.)**	45.37 ± 28.37	14.87 ± 6.76	32.52 ± 17.60
**Absolute increase**	+18.38	+5.45	+11.34
**Relative increase (%)**	+68.08	+57.80	+53.57

n is the number of 25 m x 25 m grid cells in each rangeland.

**Fig 2:**
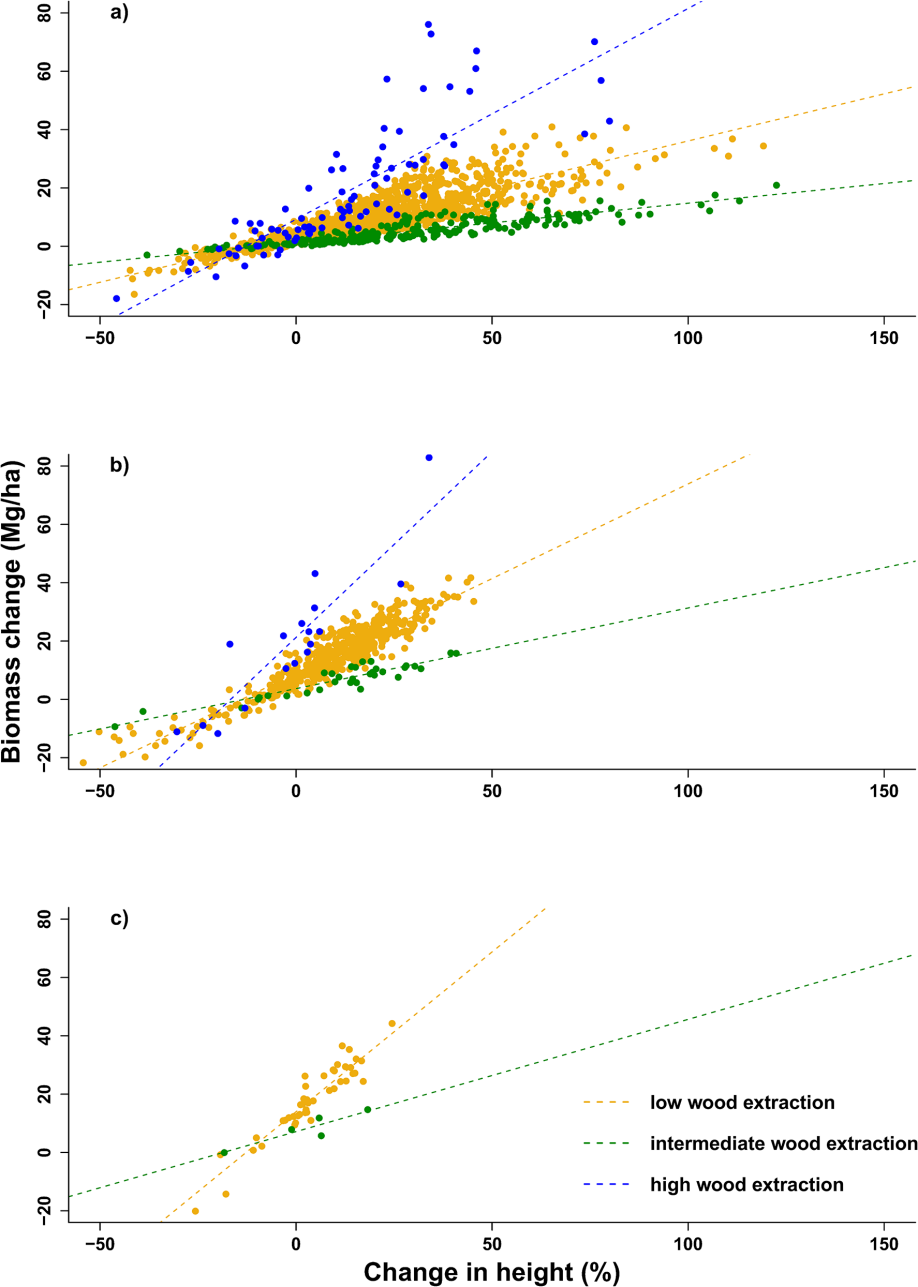
Height-specific biomass change as a function of relative height change per grid cell. Height categories are a) 1–3 m, b) 3–5 m and c) 5–10 m for rangelands of high, intermediate and low wood extraction pressure. There were no data for the 5–10 m height class in the high wood extraction rangeland and the >10 m height class for all rangelands as there were no grid cells with an average height over 10 m. Grid cell size: 25 m x 25 m.

**Fig 3:**
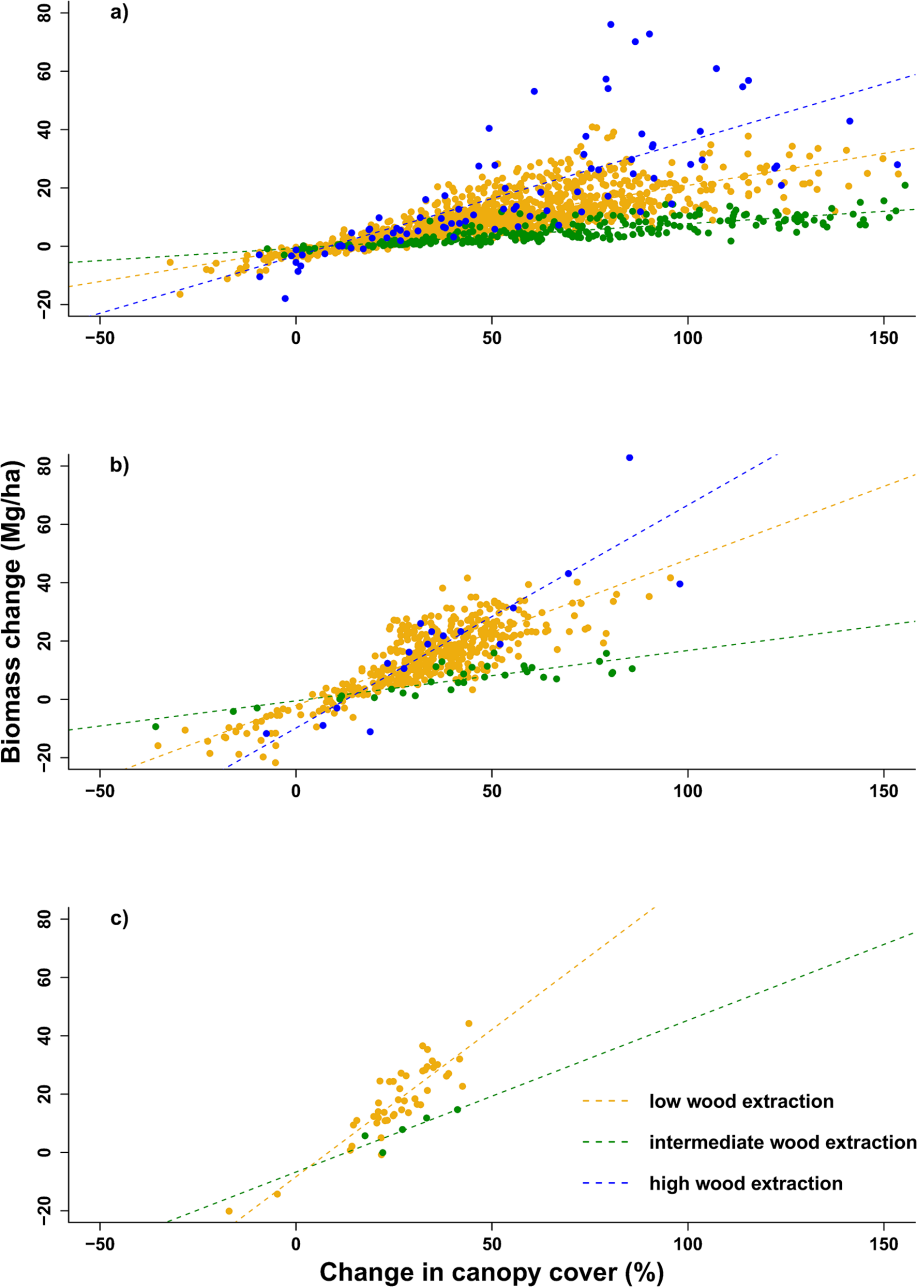
Height-specific biomass change as a function of relative change in canopy cover per grid cell. Height categories are a) 1–3 m, b) 3–5 m and c) 5–10 m for rangelands of high, intermediate and low wood extraction pressure. There were no data for the 5–10 m height class in the high wood extraction rangeland and the >10 m height class for all rangelands as there were no grid cells with an average height over 10 m. Grid cell size: 25 m x 25 m.

Variability increased with increased biomass, particularly in the high and low extraction pressure sites (Table 2). Represented as a rate of biomass change, the mean annual woody biomass productivity (± 95% spatial confidence interval) translates to 14 ± 1.39% p.a, 12 ± 0.08% p.a. and 11 ± 0.00% p.a for the high, intermediate and low wood extraction sites, respectively. These increases were despite ongoing wood harvesting in these rangelands. Relative to the starting biomass, all mean increases were greater than 50% (Table 2). Extreme biomass increases were related to large changes in relative height (Fig 2) and relative canopy cover (e.g. >50% increase in canopy cover results in biomass increases of >20 Mg ha^-1^, Fig 3). However, the extreme biomass changes (i,e. >40 Mg ha^-1^) predominantly occurred in the 1–3 m height class (Fig 2A and Fig 3A). Biomass increases of >40 Mg ha^-1^ did not occur in height classes >5 m (Fig 2C and Fig 3C). The largest increases in biomass occur in the high wood extraction site when compared with the same increases in relative height (Fig 2A and 2B) and canopy cover (Fig 3A and 3B) in the other rangelands. There are no data for the high extraction site for the 5-10m height class as there are no grid cells with an average height >5 m in this rangeland (Fig 2C and Fig 3C).

### Vegetation structural dynamics

**Fig 4:**
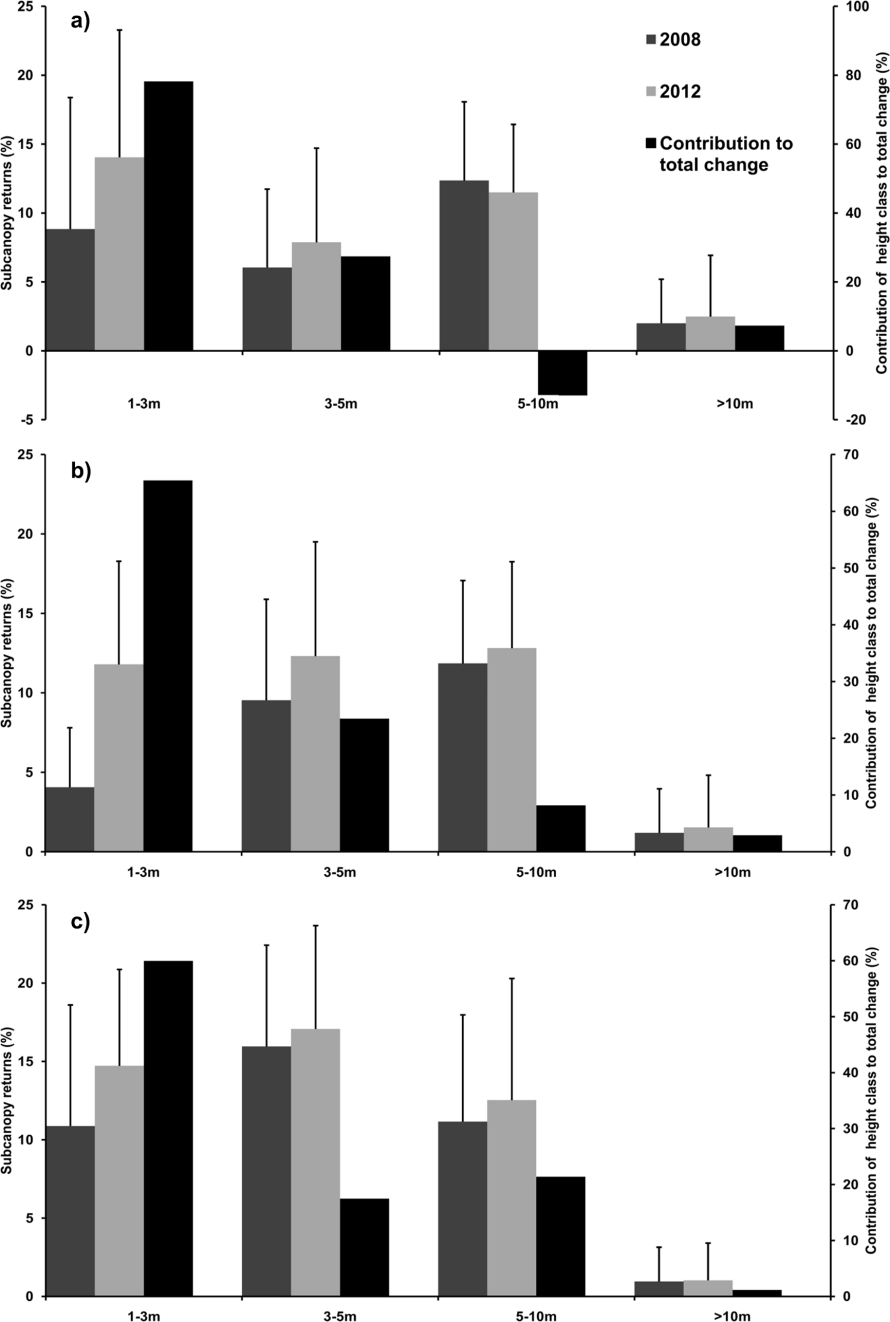
Height-specific subcanopy returns (%) (mean ± standard deviation) for 2008 and 2012. Wood extraction levels are: a) high (n = 102 cells), b) intermediate (n = 291 cells), and c) low wood extraction (n = 1654 cells). Contribution of height class change (subcanopy returns) to total change (total vegetation column) (%) is the black bar represented by values on the secondary axis. e.g. In the high wood extraction rangeland, 79% of the change in the total vegetation column was attributable to the 1–3 m height class.

Total % canopy returns increased between 2008 and 2012 in all rangelands, but up to 79% of the total change in canopy returns was attributable to the increase in the 1–3 m height category within the subcanopy (Fig 4). Losses in subcanopy returns were only found in the high wood extraction rangeland, and only in the 5–10 m height class (Fig 4A). There was little contribution to total change in % subcanopy returns from the >10 m height class (Fig 4). Although the high and low extraction rangelands had fairly similar overall increases in % total canopy returns, this was not the case with relative change (from 2008), where the highest extraction site was far greater (e.g. relative canopy returns for height class 1–3 m: 425%, 387% and 90% for high, intermediate and low extraction, respectively). Thus, the order of relative change in % canopy returns followed the gradient of wood extraction levels at the different sites.

Another indicator of shrub level increase in the rangelands is the change in the number of cells that remained after an average height mask was applied (i.e. that fulfilled the average height criteria threshold to be included in the biomass analysis), expressed as a percentage of each rangeland. The high extraction rangeland changed from 10% of the rangeland that met the average height (>1.5 m) criteria mask in 2008 to 15.9% of the rangeland in 2012 (χ^2^_1_ = 107.6; p <0.001); the intermediate use site doubled in the percentage of rangeland that met the average height criteria from 8.5% to 17.4% (χ^2^_1_ = 780.8; p <0.001); and the low use rangeland increased from 54.2% in 2008 to 63.8% of the rangeland in 2012 (χ^2^_1_ = 220.7; p <0.001).

### Association between biomass change and vegetation subcanopy returns

**Fig 5:**
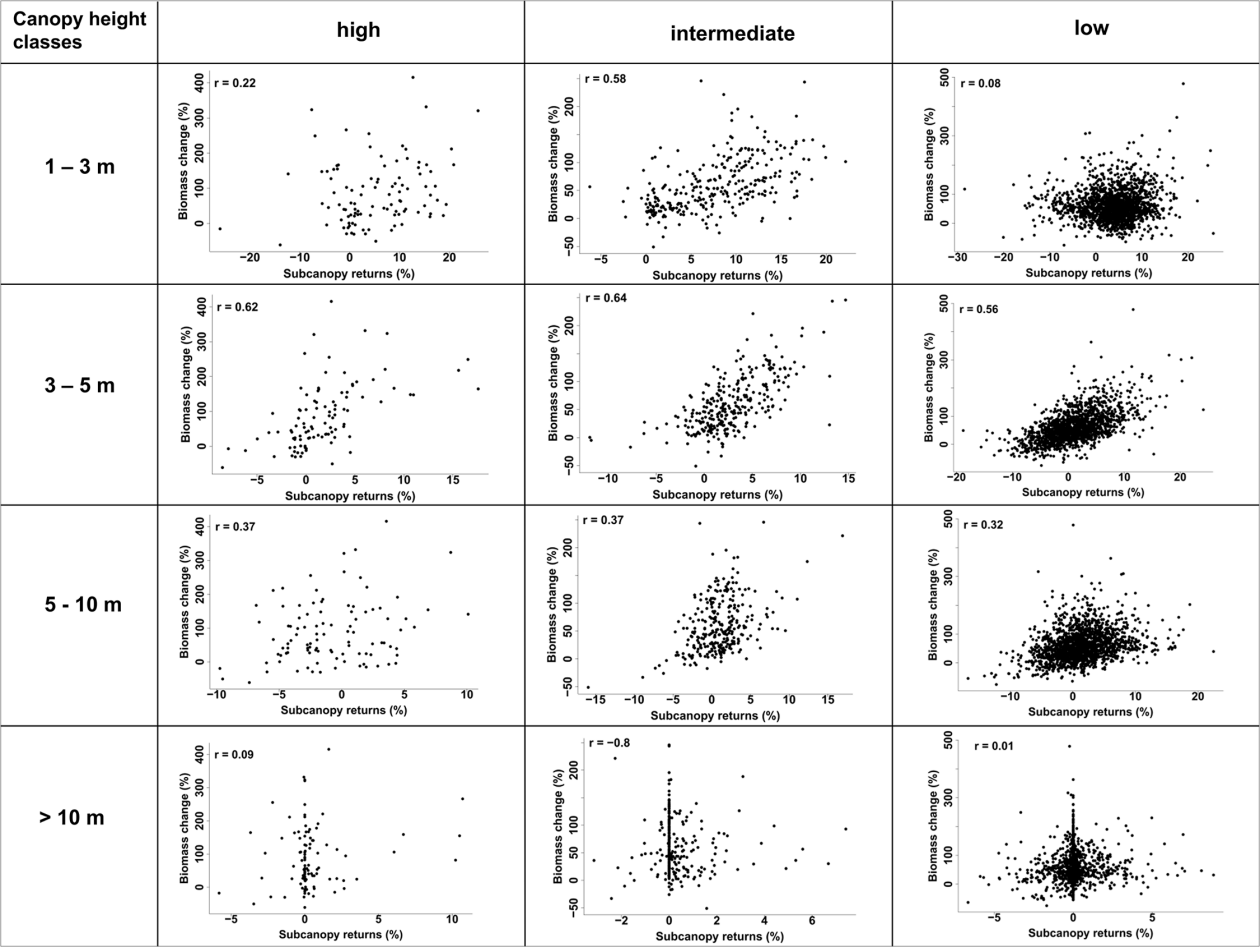
Height-specific correlation (p < 0.001) between change in biomass (%) and subcanopy returns (%). Wood extraction levels for each rangeland are listed per column as high, intermediate and low.

There was a positive correlation between change in biomass and change in % subcanopy returns (Fig 5); particularly in the 1–3 m height class in the high extraction sites (high extraction: r = 0.22, p <0.0001; intermediate extraction: r = 0.58, p<0.0001) and the 3–5 m height class (high extraction: r = 0.62, p <0.0001; intermediate extraction: r = 0.64, p <0.0001; low extraction: r = 0.56, p <0.0001). Although this relationship was also present in the 5–10 m height class at all extraction levels (r >0.31), it degraded at heights >10 m (r < 0.10) (Fig 5). It is interesting to note that the strength of the relationship between change in biomass and change in % subcanopy returns across all height categories was strongest at the intermediate wood extraction site (Fig 5).

Changes in biomass and height-specific subcanopy returns were spatially associated (S2 Fig). However, these changes were more apparent at <5 m (S2 Fig). Almost no change in % subcanopy return for vegetation >10 m is evident (S2 Fig). The same biomass values for a given grid cell can manifest as different structural profiles. As such, structural profiles could change in different ways whilst maintaining the same overall biomass value outcome. For example, if the site was dominated by grasses with several trees >5 m, that site could, theoretically, show no change in biomass value by 2012, but the structural profile may have changed to predominant shrub cover and fewer tall trees.

## Discussion

Large increases in biomass at all sites (Table 2) are in contradiction to previous fuelwood supply-demand models which predicted biomass depletion [24,32,63]. Biomass increases in Bushbuckridge rangelands were attributable (>80%) to vegetation in the 1–3 m height class within the subcanopy (Fig 4), with extreme biomass gains (>20 Mg ha^-1^) associated with vegetation that gained >25% in height (Fig 2A) or >50% in canopy cover (Fig 3A). This agrees with an observed increase in the number of thinner, taller stems within Bushbuckridge rangelands [35] and more grid cells meeting the average height criteria in each of the rangelands between 2008 and 2012. These low height class increases probably reflect local-scale dynamics of harvesting—more harvesting drives coppicing (resprouting from the stem or roots) in the intermediate and high extraction sites (Fig 2A and Fig 3A)—but the relationship appears more pronounced in the intermediate site as less of the coppice is harvested. It is likely that wood harvesting is acting as a ‘bush thinning’ mechanism, changing the size specific growth rates, particularly in resprouting from stumps with fully-developed root systems [64]. Indeed, thick stands of small-stemmed trees can yield more woody biomass than a few, large trees as a result of divergent, size-specific growth rates [65]. However, low height class increases in biomass could also be a result of newly established bush encroachers which characteristically invade overgrazed and degraded rangelands [66–68]. Biomass estimations for different height classes in a savanna woodland reveal, collectively, greater biomass quantities are located below 4.5 m in height than above; a disparity more prominent immediately after a disturbance [69]. Harvesting has been found to increase the density of smaller stems without changing the height structure of the woodland [70]. Unfortunately, there is a dearth of data on the preferred height of harvested species, only preferred diameter size which ranges, location dependent, between 2–6.5 cm [26,36]. There are records of stems >1 cm being taken, with preference for those >4 cm and almost no stems harvested >20 cm [71]. Extrapolating 1 cm and 5 cm diameter size into available coppice diameter-height allometry relationships [72] suggests pre-harvested heights of 0.74 m and 2.92 m in *Dichrostachys cinerea*, *0*.63 m and 2.07 m in *Acacia harveyi*, and 0.77 m and 2.44 m for *Combretum collinum*, respectively. Although the relationship between harvested stem diameters and regrowth shoot length is variable, we can infer that stems harvested for fuelwood are generally <3 m. Therefore, preferred ‘harvesting heights’ coincide with height class with the most subcanopy gains (Fig 4).

Subcanopy biomass increases at low heights in a rangeland context are likely a combination of woody regrowth-response (harvesting effects) [71–74] and bush encroachment (overgrazing effects) [15,75–76], here collectively referred to as ‘bush thickening’. However, these are not mutually exclusive events and can occur together. Low height-class increases occur in Bushbuckridge both as standalone shrubs as well as occurring underneath the canopies of tall trees [45]. Resprouting rates and the subsequent influence on communal rangeland dynamics have been underestimated in the earlier research in this region [77]. Although the Wessels et al. [32] supply-demand model did include resprouting estimates of 89 kg ha^-1^ yr^-1^ which is significantly higher than the 20 kg ha^-1^ yr^-1^ that the Banks et al. [24] model used; these rates are only from one species, *T*. *sericea*, and thus, may underestimate the growth rates for the other predominant coppicing species, e.g. *D*. *cinerea*. Previous data suggest that even during a poor rainfall period, in just five months there was coppice of 989 kg ha^-1^ (6.6% of the total post-harvest biomass) and harvested trees recovered two thirds of their preharvest biomass, with no harvest-induced mortality [71]. *T*. *sericea* coppice shoots from established stumps gained between 1–2 m in height over 3 years [78], whilst coppice stands in Malawi and Kenya gained 3m [79] and 2m [80], respectively, over 4 years. This is evident in the annual productivity suggested by the LiDAR-derived estimates of well over 10% p.a. (especially when we consider that this is over and above the biomass removed for wood energy) which exceeds the previous woodland productivity value of 4% [24,32,81]. The disparity in the growth rates is likely a result of higher productivity in the low height classes [69] and a significant contributor to the Wessels et al. [32] underestimation of biomass production rate. Growth rates could also have been affected by the drier than normal conditions in 2007 and, likewise, the high rainfall in 2010 and early 2012. As data collection was subsequent to these events, it is likely that biomass estimates were affected.

Although lower height classes within the subcanopy showed increases across all wood extraction sites (Fig 4), this was not true for subcanopy returns in the 5–10 m class in the high wood extraction site (Fig 4A). Large, fruiting trees are normally conserved by villagers as they are used for a variety of non-timber uses [82–83]. Despite cultural practices against live-wood harvesting of large fruiting trees, villagers acknowledge that they do cut trees, like marula (*Sclerocarya birrea*), as they feel they have no alternatives in the face of high electricity prices and localised shortages of fuelwood [83]. We observed several felled and pollarded marula trees in the highest wood extraction site and can assume, together with the lack of data for grid cells of average height >5m (Fig 2C and Fig 3C), that the loss of vegetation returns in the 5–10 m height class reflects a localised lack of fuelwood of sufficient quality and quantity in this rangeland. The reduced number of tall trees and abundance of short subcanopy vegetation in the high use rangeland results in a more homogeneous stand structure (Fig 4A), a possible explanation for the stronger relationship between field and LiDAR data in this site (Table 1). Most fuelwood supply-demand models that predicted loss of biomass are not spatially explicit and did not capture the fine scale variation at village level [84–85] or the mismatch between the spatial variability in fuelwood supply relative to centres of demand [35], especially considering vehicles are increasingly being used to transport larger amounts of wood from more distant locations [39,86]. Yet, the Wessels et al. [32] fuelwood model focused on one “best-case scenario” communal rangeland, exclusively utilised by one village and still predicted losses. However, fuelwood demand is not a linear system and people’s responses to changes in their socio-economic and natural resource environment are complex and difficult to quantify [37]; consequently, the community’s adaptive responses are not incorporated in these models. Global and national studies highlight the lack of adaptive capacity of people in the developing world [37,87–88]; however, the strategies people adopt on local and regional scales often reveal surprising resourcefulness in response to change [89–91]. Within the fuelwood context in Bushbuckridge and elsewhere in Africa, responses to localised fuelwood shortages have included: changes in the preferred size class of fuelwood [29,35,86]; switching preferred fuelwood species [25,33,91]; more frequent trips or more time spent per trip to collect fuelwood [31,92]; travelling further from home [37]; use of wheelbarrows and vehicles to collect more wood per trip [33,38,86,93]; development of fuelwood markets [33,36]; and collecting from neighbouring private land [35]. Socio-economic factors also play a role in fuelwood demand dynamics. High dependence on government social grants and migrant worker remittances is characteristic of rural areas [33,94–95]; changes in these economic flows will affect household cash flow and, thus, alter household-level demand for natural resources. These adaptive strategies and socio-economic factors are difficult to capture in a supply-demand models and are a contributing cause to the disparity between predicted and measured biomass in communal rangelands.

Biomass values range between 9 Mg ha^-1^ (on gabbro) to 27 Mg ha^-1^ (on granite) which is comparable to the range for field-based allometry studies in the greater Bushbuckridge area (18.9–23.1 Mg ha^-1^) [7], and the LiDAR-estimates for the conserved Lowveld region (11.9–92.3 Mg ha^-1^) [2]. The intermediate wood extraction site has had previous estimates of LiDAR-derived biomass for 2008 of 12 Mg ha^-1^ [32], but this used allometry from Nickless et al. [96] and field–LiDAR biomass regression relationships derived from the regional landscape. Most studies on allometry have focused on temperate zone and deciduous forests (e.g. [58,97–98]) or tropical forest monitoring (e.g. [8,99–101]). Very few have focused on savanna systems (e.g. [2,4,96]). Both Chave et al. [8] and Colgan et al. [9] stress the importance of allometric equation choice on error as even field-allometry had 16% RSE (Residual Standard Error); these errors often compound with averaging. Although Colgan et al.’s [9] plot-averaged LiDAR-derived biomass estimates had 9% more relative error (difference between predicted and measured biomass) than field-harvested biomass, the bias (mean error) was only -3% (compare to Nickless et al. [96] allometry with 15% more relative error and 50% bias) [9]. Our study also excluded all cells that were below 1.5 m in average height in both 2008 and 2012, cutting out a large proportion of the area relative to the portion used in Wessels et al.’s [32] study. Although our biomass model has fewer field-calibration sites than the Wessels et al. [32] study, our calibration sites were specific only to the area the biomass models were applied to.

While we are confident that our biomass estimates are reflecting a true increase, the shortcomings of using this method have the potential to exaggerate increases, particularly error in canopy cover measurements over time. This is of concern when considering leaf area index (LAI) in LiDAR change detection metrics, as both the voxel and the CHM data may be influenced, affecting the biomass estimates as well as the subcanopy LiDAR returns. Although this was controlled for as much as possible by collecting the LiDAR data in the same month each campaign, LAI varies with phenology and with local climatic changes, such as differential rainfall between years, or heavy winds [102]. The relatively high predictive uncertainty (RMSE range: 4.8–19.1 Mg ha^-1^) in the biomass models occur in the high and low wood extraction rangelands, both of which are situated on granitic geology (Fig 1) which are more heterogeneous in both topographic relief and stand structure, as well as in the resultant biomass (Table 1). In landscape-scale approximations of biomass, errors are introduced and often propagated. The assumption is that individual plant measurement errors will average out over the plot level, provided the plots are large enough and the measurement process is unbiased. There is also an effect of plot size on error; increasing plot size increases the predictive power of the model [10]. However, there is a trade-off between the cost and logistic realities of sampling large plots and the need to sample a large number of plots, as plot number also affects landscape-scale error [9]. Although relative uncertainty in the biomass models was high and may have been reduced by object based image analysis (OBIA) methods applied to single tree crowns to counter vertical structural heterogeneity errors, plot-level averaging methods have a positive trade-off in their simplicity and their ability to average out within-plot variation, particularly the horizontal canopy cover heterogeneity characteristic of savannas.

## Conclusions

Savanna-based biomass studies have considerable scope to rectifying the underestimation of carbon sinks and sources, elucidating the woody encroachment problem in savannas and untangling the interactions between bush encroachment/thickening and wood extraction by rural communities. Without high resolution, 3-D vegetation data covering a large area, the landscape-scale increases in biomass over the Bushbuckridge rangelands could erroneously be interpreted as woodlands recovering to an “unaltered” state. Users of two-dimensional, remotely-sensed biomass estimates should remain aware of structural implications in the landscape to make informed conclusions on vegetation dynamics, particularly in the context of increasing savanna bush encroachment in a CO_2_ rich environment [103–104]. Indeed, it is the low height class vegetation within the subcanopy which determines future woodland structure. Moreover, most carbon cycle studies in Africa neglect domestic emissions from wood harvesting [105] despite knowing the contribution of deforestation and land degradation to carbon dynamics [106]; a recent carbon model has demonstrated the importance of vegetation increases in the southern hemisphere’s semi-arid regions to terrestrial carbon sinks [107]. The repercussions of bush thickening in communal rangelands will have implications for the direct-use values of ecosystem goods and will affect household vulnerability to shocks [39]. Our research suggests that wood harvesting can, paradoxically, exacerbate bush thickening as many of the harvested savanna species have strong regenerative responses [71–72,79–80,108–109]. Not only is coppice an important survival strategy for regenerating woodlands, the resprouted stems may provide a valuable source of future harvestable biomass [74,78,110–112]. There is, however, little information on regrowth rates and response to continued harvesting as well as whether the coppice is of appropriate quality for fuelwood.

## Supporting Information

S1 Dataset Biomass model data. Data include 2012 LiDAR-derived average height and canopy cover extraction metrics, as well as field-work based allometry. Each line item is per 25 m x 25 m grid cell. Metadata are included in the dataset.
(XLSX)

S2 Dataset Biomass and subcanopy data. Data include 2008 and 2012 biomass estimates derived from biomass models as well as % subcanopy returns for voxel data for the height class categories: 1-3m, 3-5m, 5-10m and >10m. Each line item is per 25 m x 25 m grid cell. Data are organized per land extraction category into separate worksheets. Metadata are included in the dataset.
(XLSX)

S3 Dataset Biomass changes (Mg ha^-1^) in relation to relative height and canopy cover change. Data include biomass change estimates (2008–2012), percentage height and canopy cover changes for each 25 m x 25 m grid cell. Each height class (relative to height in 2008) are shown on separate worksheets. Metadata are included in the dataset.
(XLSX)

S1 Fig Site-specific biomass model residuals. The residual spread demonstrates heteroskedasticity with increasing biomass fitted values for rangelands with a) high, b) intermediate and c) low extraction pressure.
(TIFF)

S2 Fig Biomass changes (%) relative to height-specific change in subcanopy returns (%). Height categories are: 1–3 m, 3–5 m, 5–10 m and >10 m.
(TIF)

## References

[1] De Castilho CV, Magnusson WE, de Araújo RNO, Luizão RCC, Luizão FJ, Lima AP, et al Variation in aboveground tree live biomass in a central Amazonian Forest: Effects of soil and topography. For Ecol Manage. 2006;234: 85–96.

[2] Colgan MS, Asner GP, Levick SR, Martin RE, Chadwick OA. Topo-edaphic controls over woody plant biomass in South African savannas. Biogeosciences. 2012;9: 957–987. 10.5194/bgd-9-957-2012 22949722

[3] Dahlin KM, Asner GP, Field CB. Environmental filtering and land-use history drive patterns in biomass accumulation in a mediterranean-type landscape. Ecol Appl. 2012;22: 104–118. 22471078 10.1890/11-1401.1

[4] De Castro EA, Kauffman JB. Ecosystem structure in the Brazilian Cerrado: a vegetation gradient of aboveground biomass, root mass and consumption by fire. J Trop Ecol. 1998;14: 263–283.

[5] Chambers JQ, Higuchi N, Teixeira LM, dos Santos J, Laurance SG, Trumbore SE. Response of tree biomass and wood litter to disturbance in a Central Amazon forest. Oecologia. 2004;141: 596–611. 15365808 10.1007/s00442-004-1676-2

[6] Frolking S, Palace MW, Clark DB, Chambers JQ, Shugart HH, Hurtt GC. Forest disturbance and recovery: A general review in the context of spaceborne remote sensing of impacts on aboveground biomass and canopy structure. J Geophys. Res 2009;114: G00E02.

[7] Shackleton CM, Scholes RJ. Above ground woody community attributes, biomass and carbon stocks along a rainfall gradient in the savannas of the central lowveld, South Africa. South African J Bot. 2011;77: 184–192.

[8] Chave J, Andalo C, Brown S, Cairns MA, Chambers JQ, Eamus D, et al Tree allometry and improved estimation of carbon stocks and balance in tropical forests. Oecologia. 2005;145: 87–99. 15971085 10.1007/s00442-005-0100-x

[9] Colgan MS, Asner GP, Swemmer AM. Harvesting tree biomass at the stand-level to assess the accuracy of field and airborne biomass estimation in savannas. Ecol Appl. 2013;23: 1170–1184. 23967584 10.1890/12-0922.1

[10] Asner GP, Mascaro J. Mapping tropical forest carbon: Calibrating plot estimates to a simple LiDAR metric. Remote Sens Environ. 2014;140: 614–624.

[11] Fisher JT, Erasmus BFN, Witkowski ETF, van Aardt J, Wessels KJ, Asner GP. Savanna woody vegetation classification—now in 3-D. Appl Veg Sci. 2014.

[12] Sankaran M, Hanan NP, Scholes RJ, Ratnam J, Augustine DJ, Cade BS, et al Determinants of woody cover in African savannas. Nature. 2005;438: 846–849. 16341012 10.1038/nature04070

[13] Sankaran M, Ratnam J, Hanan N. Woody cover in African savannas: the role of resources, fire and herbivory. Glob Ecol Biogeogr. 2008;17: 236–245.

[14] Bond WJ, Midgley GF. Carbon dioxide and the uneasy interactions of trees and savannah grasses. Philos Trans R Soc Lond B Biol Sci. 2012;367: 601–612. 10.1098/rstb.2011.0182 22232770 PMC3248705

[15] Scholes RJ, Archer SR. Tree-Grass Interactions in Savannas. Annu Rev Ecol Syst. 1997;28: 517–544.

[16] Higgins SI, Bond WJ, Trollope WSW. Fire, resprouting and variability: a recipe for grass-tree in savanna coexistence. J Ecol. 2000;88: 213–229.

[17] Bond WJ, Keeley JE. Fire as a global “herbivore”: the ecology and evolution of flammable ecosystems. Trends Ecol Evol. 2005;20: 387–394. 16701401 10.1016/j.tree.2005.04.025

[18] Smit IPJ, Asner GP, Govender N, Kennedy-Bowdoin T, Knapp DE, Jacobson J. Effects of fire on woody vegetation structure in African savanna. Ecol Appl. 2010;20: 1865–1875. 21049875 10.1890/09-0929.1

[19] Staver AC, Archibald S, Levin S. Tree cover in sub-Saharan Africa: rainfall and fire constrain forest and savanna as alternative stable states. Ecology. 2001;92: 1063–1072. 10.1890/10-1684.1 21661567

[20] Bond WJ. What Limits Trees in C4 Grasslands and Savannas? Annu Rev Ecol Evol Syst. 2008;39: 641–659.

[21] Staver AC, Bond WJ, Stock WD, Van Rensburg SJ, Waldram MS. Browsing and fire interact to suppress tree density in an African savanna. Ecol Appl. 2009;19: 1909–1919. 19831079 10.1890/08-1907.1

[22] Asner GP, Levick SR. Landscape-scale effects of herbivores on treefall in African savannas. Ecol Lett. 2012;15: 1211–1217. 10.1111/j.1461-0248.2012.01842.x 22863324

[23] Archibald S, Roy DP, Van Wilgen BW, Scholes RJ. What limits fire? An examination of drivers of burnt area in Southern Africa. Glob Chang Biol. 2009;15: 613–630.

[24] Banks DJ, Griffin NJ, Shackleton CM, Shackleton SE, Mavrandonis JM. Wood supply and demand around two rural settlements in a semi-arid savanna, South Africa. Biomass and Bioenergy. 1996;11: 319–331.

[25] Luoga EJ, Witkowski ET, Balkwill K. Economics of charcoal production in miombo woodlands of eastern Tanzania: some hidden costs associated with commercialization of the resources. Ecol Econ. 2000;35: 243–257.

[26] Stringer LC, Reed MS. Land degradation assessment in southern Africa: integrating local and scientific knowledge bases. L Degrad Dev. 2007;18: 99–116.

[27] Kalema VN, Witkowski ETF. Land-use impacts on woody plant density and diversity in an African savanna charcoal production region. Int J Biodivers Sci Ecosyst Serv Manag. 2012;8: 1–17.

[28] Abbot JIO, Homewood K. A history of change: causes of miombo woodland decline in a protected area in Malawi. J Appl Ecol. 1999;36: 422–433.

[29] Furukawa T, Fujiwara K, Kiboi SK, Mutiso PBC. Threshold change in forest understory vegetation as a result of selective fuelwood extraction in Nairobi, Kenya. For Ecol Manage. 2011;262: 962–969.

[30] Scholes RJ, Biggs R. Ecosystem Services in southern Africa: A Regional Assessment. 2004: Available: http://www.unep.org/maweb/documents_sga/SAfMA_Regional_Report_-_final.pdf . Accessed 2014 Nov 18.

[31] Dovie DBK, Witkowski ETR, Shackleton CM. The fuelwood crisis in southern Africa—relating fuelwood use to livelihoods in a rural village. GeoJournal. 2004;60: 123–133.

[32] Wessels KJ, Colgan MS, Erasmus BFN, Asner GP, Twine WC, Mathieu R, et al Unsustainable fuelwood extraction from South African savannas. Environ Res Lett. 2013;8 24554969

[33] Madubansi M, Shackleton CM. Changes in fuelwood use and selection following electrification in the Bushbuckridge lowveld, South Africa. J Environ Manage. 2007;83: 416–426. 16930808 10.1016/j.jenvman.2006.03.014

[34] Shackleton CM, McConnachie M, Chauke MI, Mentz J, Sutherland F, Gambiza J, et al Urban fuelwood demand and markets in a small town in South Africa: Livelihood vulnerability and alien plant control. Int J Sustain Dev World Ecol. 2006;13: 481–491.

[35] Matsika R, Erasmus BFN, Twine WC. A tale of two villages: assessing the dynamics of fuelwood supply in communal landscapes in South Africa. Environ Conserv. 20112;40: 71–83.

[36] Twine W, Siphugu V, Moshe D. Harvesting of communal resources by “outsiders” in rural South Africa: a case of xenophobia or a real threat to sustainability? Int J Sustain Dev World Ecol. 2003;10: 263–274.

[37] Giannecchini M, Twine W, Vogel C. Land-cover change and human–environment interactions in a rural cultural landscape in South Africa. Geogr J. 2007;173: 26–42.

[38] Twine WC. Socio-economic transitions influence vegetation change in the communal rangelands of the South African lowveld. African J Range Forage Sci. 2005;22: 93–99.

[39] Twine W, Moshe D, Netshiluvhi T, Siphugu V. Consumption and direct-use values of savanna bio-resources used by rural households in Mametja, a semi-arid area of Limpopo province, South Africa. S Afr J Sci. 2003;99: 467–473.

[40] Lefsky MA, Cohen WB, Parker GG, Harding DJ. Lidar Remote Sensing for Ecosystem Studies. Bioscience. 2002;52: 19–30.

[41] Herremans M. Effects of woodland modification by African elephant *Loxodonta africana* on bird diversity in northern Botswana. Ecography. 1995;18: 440–454.

[42] Halaj J, Ross DW, Moldenke AR. Importance of Habitat Structure to the Arthropod Food-Web in Douglas-Fir Canopies. Oikos. 2000;90: 139–152.

[43] Lumsden LF, Bennett AF. Scattered trees in rural landscapes: foraging habitat for insectivorous bats in south-eastern Australia. Biol Conserv. 2005;122: 205–222.

[44] Hall FG, Bergen K, Blair JB, Dubayah R, Houghton R, Hurtt G, et al Characterizing 3D vegetation structure from space: Mission requirements. Remote Sens Environ. 2011;115: 2753–2775.

[45] Fisher JT, Witkowski ETF, Erasmus BFN, Mograbi PJ, Asner GP, van Aardt JAN, et al. What lies beneath: Detecting sub-canopy changes in savanna woodlands using a three-dimensional classification method. Appl Veg Sci: 2015.

[46] Goatley CHR, Bellwood DR. The Roles of Dimensionality, Canopies and Complexity in Ecosystem Monitoring. PLoS One. 2011;6: e27307:1–8. 10.1371/journal.pone.0027307 22073311 PMC3207849

[47] Rutherford M, Mucina L, Lotter MC, Bredenkamp GJ, Smit JHL, Scott-Shaw CR, et al Savanna biome In: Mucina L, Rutherford MC, editors. The Vegetation of South Africa, Lesotho and Swaziland. 2006: Pretoria, South Africa: South African National Biodiversity Institute pp. 439–539.

[48] Coetzer KL, Erasmus BFN, Witkowski ETF, Bachoo AK. Land-cover change in the Kruger to Canyons Biosphere Reserve (1993–2006): A first step towards creating a conservation plan for the subregion. S Afr J Sci. 2010;106: 1–10.

[49] Pollard S, Du Toit D, Biggs H. River management under transformation: The emergence of strategic adaptive management of river systems in the Kruger National Park. Koedoe—African Prot Area Conserv Sci. 2011;53: 1–14. 7252963

[50] Thornton R. Environment and Land in Bushbuckridge, South Africa In: Zarsky L, editor. Human Rights & the Environment: Conflicts and Norms in a Globalizing World. 2002: London, U.K.: Earthscan Publications pp. 219–240.

[51] Shackleton CM. Comparison of plant diversity in protected and communal lands in the Bushbuckridge lowveld savanna, South Africa. Biol Conserv. 2000;94: 273–285.

[52] Coetzer KL, Erasmus BFN, Witkowski ETF, Reyers B. The Race for Space: Tracking Land-Cover Transformation in a Socio-ecological Landscape, South Africa. Environ Manage. 2013;52: 595–611. 10.1007/s00267-013-0094-9 23811775

[53] Fisher JT, Witkowski ETF, Erasmus BFN, Van Aardt J, Asner GP, Wessels KJ, et al Human-modified landscapes: patterns of fine-scale woody vegetation structure in communal savannah rangelands. Environ Conserv. 2011;39: 72–82.

[54] Asner GP, Knapp DE, Kennedy-Bowdoin T, Jones MO, Martin RE, Boardman J, et al Carnegie Airborne Observatory: in-flight fusion of hyperspectral imaging and waveform light detection and ranging for three-dimensional studies of ecosystems. J Appl Remote Sens. 2007;1: 1–21.

[55] Asner GP, Knapp DE, Boardman J, Green RO, Kennedy-Bowdoin T, Eastwood M, et al Carnegie Airborne Observatory-2: Increasing science data dimensionality via high-fidelity multi-sensor fusion. Remote Sens Environ. 2012;124: 454–465.

[56] Weishampel JF, Blair JB, Knox RG, Dubayah R, Clark DB. Volumetric lidar return patterns from an old-growth tropical rainforest canopy. Int J Remote Sens. 2000;21: 409–415.

[57] Lefsky MA, Cohen WB, Harding DJ, Parker GG, Acker SA, Gower T. Lidar remote sensing of above-ground biomass in three biomes. Glob Ecol Biogeogr. 2002;11: 393–399.

[58] Popescu SC. Estimating biomass of individual pine trees using airborne lidar. Biomass and Bioenergy. 2007;31: 646–655.

[59] Fischer J, Lindenmayer DB, Manning AD. Biodiversity, Ecosystem Function, and Resilience: Ten Guiding Principles for Commodity Production Landscapes. Front Ecol Environ. 2006;4: 80–86.

[60] Tews J, Brose U, Grimm V, Tielbörger K, Wichmann MC, Schwager M, et al Animal species diversity driven by habitat heterogeneity/diversity: the importance of keystone structures. J Biogeogr. 2004;31: 79–92.

[61] Wessels KJ, Mathieu R, Erasmus BFN, Asner GP, Smit IPJ, van Aardt JAN, et al Impact of communal land use and conservation on woody vegetation structure in the Lowveld savannas of South Africa. For Ecol Manage. 2011;261: 19–29.

[62] Parresol BR. Assessing Tree and Stand Biomass: A Review with Examples and Critical Comparisons. For Sci. 1999;4: 573–593.

[63] De Montalembert MR, Clement J. Fuelwood supplies in developing countries Food Agric Organ United States 1983: Rome, Italy, Forestry Paper No. 42. Available: http://www.fao.org/docrep/x5329e/x5329e00.HTM. Accessed 2014 Nov 18.

[64] Harrington RA, Fownes JH. Radiation interception and growth of planted and coppice stands of four fast-growing tropical trees. J Appl Ecol. 1995;32: 1–8.

[65] Caspersen JP, Vanderwel MC, Cole WG, Purves DW. How stand productivity results from size- and competition-dependent growth and mortality. PLoS One. 2011;6: e28660:1–12. 10.1371/journal.pone.0028660 22174861 PMC3236764

[66] Archer S. Have southern Texas savannas been converted to woodlands in recent history? Am Nat. 1989;134: 545–561.

[67] Moleele NM, Ringrose S, Matheson W, Vanderpost C. More woody plants? The status of bush encroachment in Botswana’s grazing areas. J Environ Manage. 2002;64: 3–11. 11876072 10.1006/jema.2001.0486

[68] Wigley BJ, Bond WJ, Hoffman MT. Bush encroachment under three contrasting land-use practices in a mesic South African savanna. Afr J Ecol. 2009;47: 62–70.

[69] Shackleton CM, Scholes RJ. Impact of fire frequency on woody community structure and soil nutrients in the Kruger National Park. Koedoe. 2000;43: 75–81.

[70] Gaugris JY, Van Rooyen MW. Woody vegetation structure in conserved versus communal land in a biodiversity hotspot: A case study in Maputaland, South Africa. South African J Bot. 2010;76: 289–298.

[71] Neke KS, Owen-Smith N, Witkowski ETF. Comparative resprouting response of Savanna woody plant species following harvesting: the value of persistence. For Ecol Manage. 2006;232: 114–123.

[72] Kaschula S, Twine W, Scholes M. The effect of catena position and stump characteristics on the coppice response of three savannah fuelwood species. Environ Conserv. 2005;32: 76–84.

[73] Kennedy AD. Coppicing of *Tarconanthus camphoratus* (Compositae) as a source of sustainable fuelwood production: an example from the Laikipia Plateau, Kenya. Afr J Ecol. 1998;36: 148–158.

[74] Shackleton CM. Stump size and the number of coppice shoots for selected savanna tree species. South African J Bot. 2000;66: 124–127.

[75] Miller RF, Wigand PE. Holocene Changes in Semiarid Woodlands Response to climate, fire, and human activities in the. Bioscience. 1994;44: 465–474.

[76] Archer S, Schimel DS, Holland EA. Mechanisms of shrubland expansion: land use, climate or CO2? Clim Change. 1995;29: 91–99.

[77] Higgins SI, Shackleton CM, Robinson ER. Changes in woody community structure and composition under constrasting landuse systems in a semi-arid savanna, South Africa. J Biogeogr. 1999;26: 619–627.

[78] Shackleton CM. Managing regrowth of an indigenous savanna tree species (Terminalia sericea) for fuelwood: the influence of stump dimensions and post-harvest coppice pruning. Biomass and Bioenergy. 2001;20: 261–270.

[79] Abbot PG, Lowore JD. Characteristics and management potential of some indigenous firewood species in Malawi. For Ecol Manage. 1999;119: 111–121.

[80] Okello BD, O’Connor TG, Young TP. Growth, biomass estimates, and charcoal production of *Acacia drepanolobium* in Laikipia, Kenya. For Ecol Manage. 2001;142: 143–153.

[81] Rutherford MC. Primary production ecology in southern Africa In: Werger MJA, editor. Biogeography and Ecology of Southern Africa. 1978: The Hague: Junk pp. 621–660.

[82] Shackleton CM, Botha J, Emanuel PL. Productivity and Abundance of *Sclerocarya birrea* Subsp. *caffra* in and Around Rural Settlements and Protected Areas of the Bushbuckridge Lowveld, South Africa. For Trees Livelihoods. 2003;13: 217–232.

[83] Kirkland T, Hunter LM, Twine W. “The Bush is No More”: Insights on Institutional Change and Natural Resource Availability in Rural South Africa. Soc Nat Resour. 2007;20: 337–350. 21909188 10.1080/08941920601161353 PMC3170092

[84] Masera O, Ghilardi A, Drigo R, Angel Trossero M. WISDOM: A GIS-based supply demand mapping tool for woodfuel management. Biomass and Bioenergy. 2006;30: 618–637.

[85] Top N, Mizoue N, Ito S, Kai S. Spatial analysis of woodfuel supply and demand in Kampong Thom Province, Cambodia. For Ecol Manage. 2004;194: 369–378.

[86] Luoga EJ, Witkowski ET, Balkwill K. Harvested and standing wood stocks in protected and communal miombo woodlands of eastern Tanzania. For Ecol Manage. 2002;164: 15–30.

[87] Schneider SH, Semenov S, Patwardhan A, Burton I, Magadza CHD, Oppenheimer M, et al Assessing Key Vulnerabilities and the Risk from Climate Change In: Parry ML, Canziani OF, Palutikof JB, van der Linden PJ, Hanson CE, editors. Climate Change 2007: Impacts, Adaptation and Vulnerability. Contribution of Working Group II to the Fourth Assessment Report of the Intergovernmental Panel on Climate Change. 2007: Cambridge, U.K.: Cambridge University Press Available: http://www.ipcc.ch/publications_and_data/ar4/wg2/en/ch19s19-es.html. Accessed 2014 Nov 18.

[88] Beg N, Morlot JC, Davidson O, Afrane-Okesse Y, Tyani L, Denton F, et al Linkages between climate change and sustainable development. Clim Policy. 2002;2: 129–144.

[89] Mortimore MJ, Adams WM. Farmer adaptation, change and “crisis” in the Sahel. Glob Environ Chang. 2001;11: 49–57.

[90] Thomas DSG, Twyman C. Equity and justice in climate change adaptation amongst natural-resource-dependent societies. Glob Environ Chang. 2005;15: 115–124.

[91] Arnold M, Kohlin G, Persson R, Shepherd G. Fuelwood Revisited: What Has Changed in the Last Decade? 2003: Available: http://www.cifor.cgiar.org/publications/pdf_files/OccPapers/OP-39.pdf. Accessed 2015 Feb 28.

[92] Matsika R, Erasmus BFN, Twine WC. Double jeopardy: The dichotomy of fuelwood use in rural South Africa. Energy Policy. 2013;52: 716–725.

[93] Vasconcelos MJP, Mussá Biai JC, Araújo A, Diniz MA. Land cover change in two protected areas of Guinea-Bissau (1956–1998). Appl Geogr. 2002;22: 139–156.

[94] Shackleton CM, Guthrie G, Main R. Estimating the potential role of commercial over-harvesting in resource viability: a case study of five useful tree species in South Africa. L Degrad Dev. 2005;16: 273–286.

[95] Butz RJ. Changing land management: A case study of charcoal production among a group of pastoral women in northern Tanzania. Energy Sustain Dev. 2013;17: 138–145.

[96] Nickless A, Scholes RJ, Archibald S. A method for calculating the variance and confidence intervals for tree biomass estimates obtained from allometric equations. South African J Sci Sci. 2011;107: 86–95.

[97] Patenaude G, Hill R, Milne R, Gaveau DLA, Briggs BBJ, Dawson TP. Quantifying forest above ground carbon content using LiDAR remote sensing. Remote Sens Environ. 2004;93: 368–380.

[98] Anderson J, Martin ME, Smith M-L, Dubayah RO, Hofton MA, Hyde P, et al The use of waveform lidar to measure northern temperate mixed conifer and deciduous forest structure in New Hampshire. Remote Sens Environ. 2006;105: 248–261.

[99] Drake JB, Dubayah RO, Clark DB, Knox RG, Blair JB, Hofton MA, et al Estimation of tropical forest structural characteristics using large-footprint lidar. Remote Sens Environ. 2002;79: 305–319.

[100] Asner GP, Flint Hughes R, Varga TA., Knapp DE, Kennedy-Bowdoin T. Environmental and Biotic Controls over Aboveground Biomass Throughout a Tropical Rain Forest. Ecosystems. 2009;12: 261–278.

[101] Asner GP, Mascaro J, Muller-Landau HC, Vieilledent G, Vaudry R, Rasamoelina M, et al A universal airborne LiDAR approach for tropical forest carbon mapping. Oecologia. 2012;168: 1147–1160. 10.1007/s00442-011-2165-z 22033763

[102] Ryu Y, Verfaillie J, Macfarlane C, Kobayashi H, Sonnentag O, Vargus R, et al Continuous observation of tree leaf area index at ecosystem scale using upward-pointing digital cameras. Remote Sens Environ. 2012;126: 116–125.

[103] Kgope BS, Bond WJ, Midgley GF. Growth responses of African savanna trees implicate atmospheric [CO2] as a driver of past and current changes in savanna tree cover. Austral Ecol. 2009;35: 451–463.

[104] Buitenwerf R, Bond WJ, Stevens N, Trollope WSW. Increased tree densities in South African savannas: >50 years of data suggests CO_2_ as a driver. Glob Chang Biol. 2012;18: 675–684.

[105] Williams CA, Hanan NP, Neff JC, Scholes RJ, Berry JA, Denning AS, et al Africa and the global carbon cycle. Carbon Balance Manag. 2007;2: 1–13. 10.1186/1750-0680-2-3 PMC1821324 17343752

[106] Denman KL, Brasseur G, Chidthaisong A, Ciais P, Cox PM, Dickinson RE, et al Couplings Between Changes in the Climate System and Biogeochemistry In: Solomon S, Qin D, Manning M, Chen Z, Marquis M, Averyt KB, et al, editors. The Physical Science Basis. Contribution of Working Group I to the Fourth Assessment Report of the Intergovernmental Panel on Climate Change. 2007: Cambridge, U.K.: Cambridge University Press Available: http://www.ipcc.ch/publications_and_data/ar4/wg1/en/ch7.html. Accessed 2014 Nov 18.

[107] Poulter B, Frank D, Ciais P, Myneni RB, Andela N, Bi J, et al. Contribution of semi-arid ecosystems to interannual variability of the global carbon cycle. Nature. 2014. 10.1038/nature13376 24847888

[108] Luoga EJ, Witkowski ETF, Balkwill K. Regeneration by coppicing (resprouting) of miombo (African savanna) trees in relation to land use. For Ecol Manage. 2004;189: 23–35.

[109] Mwavu EN, Witkowski ETF. Sprouting of woody species following cutting and tree-fall in a lowland semi-deciduous tropical rainforest, North-Western Uganda. For Ecol Manage. 2008;255: 982–992.

[110] Shackleton C. Fuelwood harvesting and sustainable utilisation in a communal grazing land and protected area of the eastern Transvaal lowveld. Biol Conserv. 1993;63: 247–254.

[111] Kaschula SA, Twine WE, Scholes MC. Coppice Harvesting of Fuelwood Species on a South African Common: Utilizing Scientific and Indigenous Knowledge in Community Based Natural Resource Management. Hum Ecol. 2005;33: 387–418.

[112] Moyo H, Scholes MC, Twine W. The effects of repeated cutting on coppice response of Terminalia sericea. Trees. 2014:

