# Supplementary figures and images for: Biomass Increases Go under Cover: Woody Vegetation Dynamics in South African Rangelands
Source: PLoS One. 2015 May 13;10(5):e0127093. doi: 10.1371/journal.pone.0127093 (PMC4430526; doi:10.1371/journal.pone.0127093)

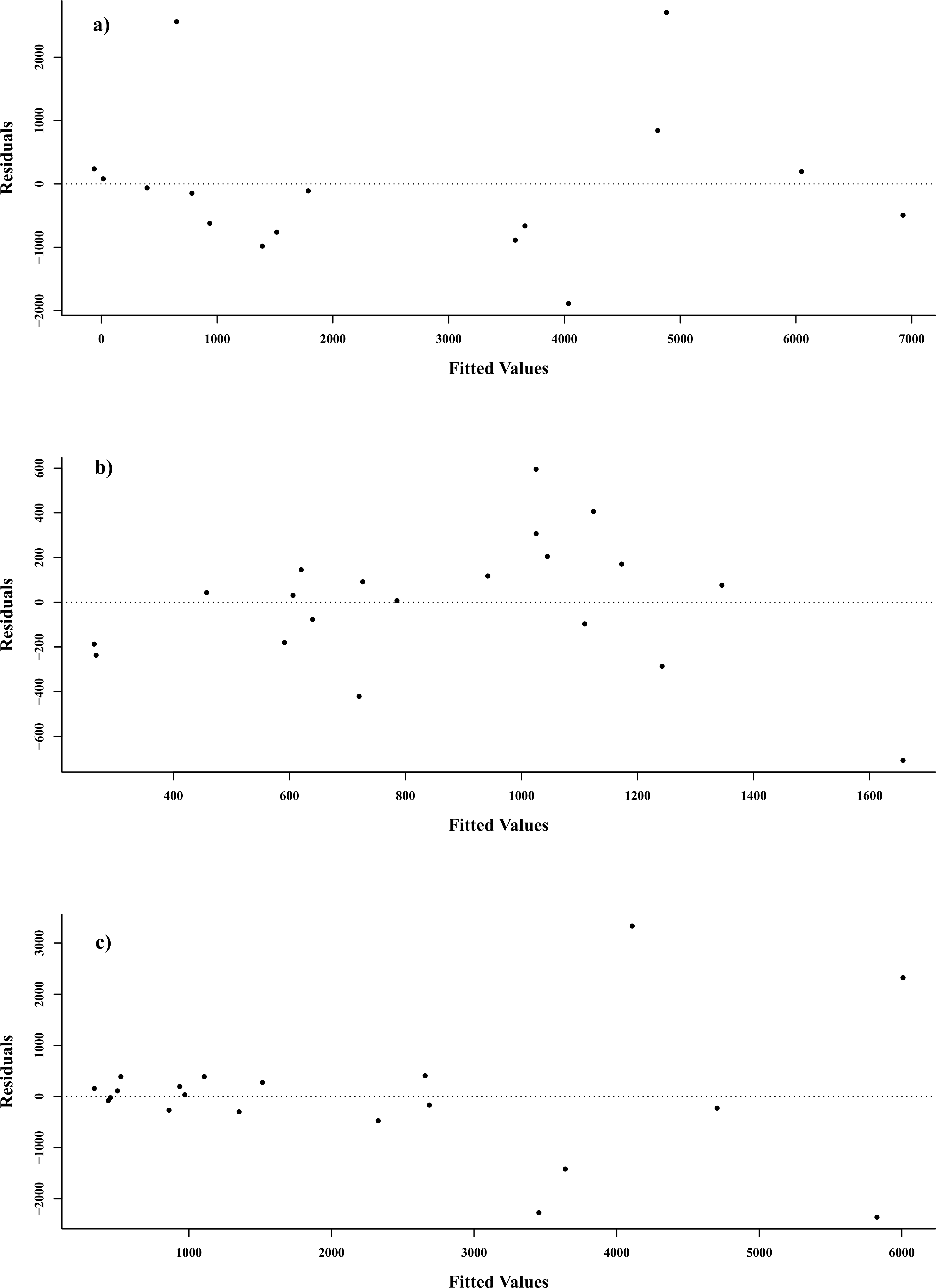

Supplement: S1 Fig — The residual spread demonstrates heteroskedasticity with increasing biomass fitted values for rangelands with a) high, b) intermediate and c) low extraction pressure. (TIFF) [file pone.0127093.s004.tiff]
